# Linoleic acid drives pulmonary lymphoepithelioma-like carcinoma progression via PPAR-α/TF axis

**DOI:** 10.3389/fonc.2025.1640201

**Published:** 2025-08-15

**Authors:** Hejing Bao, Jiani Zhang, Zhuoyan Chen, Yuhuan Wang, Zhe Wang, Zhiting Chen, Ting Jiang, Baishen Zhang, Wen Zeng, Hehong Bao, Shudong Ma

**Affiliations:** ^1^ Department of Oncology, The Affiliated Panyu Center Hospital, Guangzhou Medical University, Guangzhou, Guangdong, China; ^2^ Department of Oncology, Nanfang Hospital, Southern Medical University, Guangzhou, Guangdong, China; ^3^ Cancer Institute of Panyu District, The Affiliated Panyu Center Hospital, Guangzhou Medical University, Guangzhou, Guangdong, China; ^4^ Department of Oncology, Sun Yat-sen University Cancer Centre, State Key Laboratory of Oncology in South China, Guangzhou, Guangdong, China; ^5^ Oncology Research Institute, Ganzhou Cancer Hospital, Gannan Medical University, Ganzhou, Jiangxi, China; ^6^ Department of Psychosomatic Medicine, Chongqing University Three Gorges Hospital/Chongqing Three Gorges Central Hospital, Chongqing, China

**Keywords:** primary pulmonary lymphoepithelioma-like carcinoma, multiomics, linoleic acid, tissue factor, PPAR-α

## Abstract

**Background:**

Primary pulmonary lymphoepithelioma-like carcinoma (pLELC) is a rare subtype of non-small cell lung cancer(NSCLC) with unclear etiological mechanisms. This study aimed to investigate the underlying molecular mechanisms and therapeutic targets for pLELC.

**Methods:**

Retrospectively collected samples from advanced pLELC patients underwent proteomic and metabolomic analyses, and patient-derived xenograft (PDX) models were established for validation. Data-independent acquisition (DIA) quantitative proteomics revealed upregulated tissue factor (TF) protein expression in pLELC, while untargeted metabolomics identified key metabolites such as linoleic acid (LA).

**Results:**

Results demonstrated that LA promotes tumor progression by facilitating M2-type tumor-associated macrophage infiltration and suppressing natural killer (NK) cell activity, effects reversible by the TF inhibitor Tisotumab. Mechanistic studies indicated that LA enhances TF expression via peroxisome proliferator-activated receptor α (PPAR-α), and TF inhibitors effectively counteract LA-induced malignant phenotypes.

**Conclusion:**

This study reveals that LA remodels the pLELC tumor microenvironment through the PPAR-α/TF axis, suggesting TF as a potential therapeutic target for pLELC.

## Introduction

Lymphoepithelial carcinoma is a rare epithelial-origin tumor most commonly occurring in the nasopharynx, but it can also arise in diverse anatomical sites including the lung, thymus, stomach, liver, cervix, and bladder (1–11). Primary pulmonary lymphoepithelioma-like carcinoma accounts for only 0.4% of all primary lung cancers and 0.9% of NSCLC (12, 13). Bégin et al. first reported a case of pLELC in 1987 (14). In the 2021 World Health Organization (WHO) classification of lung tumors, this disease was reclassified from the “other unclassified carcinomas” category (4th edition) to lymphoepithelial carcinoma, specifically designated as a subtype of squamous cell carcinoma. Its characteristic pathological features include diffuse strong positivity for CK5/6, p40, and p63, a prominent syncytial growth pattern, variable degrees of lymphoplasmacytic infiltration, and a strong association with Epstein-Barr virus (EBV) (1, 2).

Studies indicate that pLELC shares genetic similarities with nasopharyngeal carcinoma but exhibits significant differences from other lung cancer types, NK/T-cell lymphoma, or EBV-associated gastric carcinoma. This tumor displays a low somatic mutation frequency but exhibits widespread copy number variations. A core aspect of the host-virus interplay involves mutations and frequent deletions in type I interferon genes (15). Reported genetic alterations in pLELC include various somatic mutations and genomic abnormalities. EBV predominantly integrates into intergenic and intronic regions, with two specific miR-BamH1-A rightward transcripts (Barts), Bart5-3p and BART20-3P, showing significant upregulation (6).

Current research on pLELC has primarily focused on genomic and transcriptomic analyses, as well as EBV integration studies. However, the pathogenesis of pLELC at the proteomic and metabolomic levels remains unclear. Furthermore, research on the pLELC immune microenvironment is still limited, largely due to the frequent diagnosis at advanced stages and the difficulty in obtaining adequate tissue specimens.

## Methods

### Proteomic analysis

Serum samples from 10 subjects (5 pLELC patients and 5 healthy controls) were collected for proteomic profiling. For each subject, 7 mL of peripheral blood was drawn into coagulation tubes one day prior to treatment initiation, centrifuged at 1,000×g for 10 min at 4°C, aliquoted, and stored at −80°C. Subsequent analyses were performed by HuiJun Biotechnology Co.,Ltd. using DIA technology. MS1 and MS2 data were randomly acquired, with iRT kit peptides (Ki3002, Biognosys AG, Switzerland) spiked into all samples for retention time calibration. Spectronaut 16 software (Biognosys AG, Switzerland) was employed for data normalization and relative quantification, as previously described (16). Differential protein screening criteria included: ≥2 unique peptides; coefficient of variation (CV) < 0.5; P < 0.05; and average fold-change thresholds (AVG ≥ 1.2 for upregulation; AVG ≤ 0.83 for downregulation).

### Metabolomic profiling

Twenty-five serum samples (15 pLELC patients, 10 controls) were analyzed. Frozen samples (−80°C) were thawed on ice, vortexed for 10 sec, and 50 μL aliquots were mixed with 300 μL of ice-cold extraction solvent (acetonitrile:methanol = 1:4, v/v) containing internal standards. After vortexing for 3 min, samples were centrifuged at 12,000 rpm for 10 min at 4°C. A 200 μL supernatant aliquot was held at −20°C for 30 min, recentrifuged, and 180 μL was subjected to LC-MS analysis. Chromatographic conditions: Waters ACQUITY UPLC HSS T3 C18 column (1.8 μm, 2.1 × 100 mm); column temperature 40°C; flow rate 0.4 mL/min; injection volume 2 μL; mobile phase: water (0.1% formic acid)-acetonitrile gradient (0 min: 95:5; 11.0 min: 10:90; 12.0 min: 10:90; 12.1 min: 95:5; 14.0 min: 95:5). Raw data were converted to mzML format using ProteoWizard, followed by peak extraction, alignment, and retention time correction via XCMS. Peak areas were normalized using support vector regression (SVR), and features with <50% detection rate in any group were excluded. Metabolites were identified by matching against in-house and public databases (e.g., metDNA). Differential metabolites were screened using: variable importance in projection (VIP) ≥ 1; P < 0.05; fold change ≥ 2 or ≤ 0.5. Unsupervised Principal Component Analysis(PCA) (unit variance-scaled data) was performed using the R prcomp function, and heatmaps with Pearson correlation coefficients were generated via the Complex Heatmap package. Kyoto Encyclopedia of Genes and Genomes (KEGG) annotation and enrichment analyses were conducted for differential metabolites (17, 18).

### Immunofluorescence and immunohistochemistry

Formalin-fixed paraffin-embedded (FFPE) sections underwent antigen retrieval in citrate buffer (pH 6.0) by boiling for 15 min. Endogenous peroxidase was blocked with 3% H_2_O_2_, followed by 30 min blocking at 37°C with normal goat serum. Primary antibodies against TF (Bioss, #P13726, 1:50), CD68 (PTMBIO, #JMMR-2659, 1:100), and CD206 (PTMBIO, #PTM-5343, 1:100) were applied and incubated overnight at 4°C. Corresponding secondary antibodies—DyLight 488-conjugated goat anti-rabbit IgG (#A23220, 1:50), DyLight 488-conjugated goat anti-mouse IgG (#A23210, 1:50), and DyLight 649-conjugated goat anti-rabbit IgG (#A23630, 1:50)—were incubated in the dark at 37°C for 90 min. Nuclei were counterstained with DAPI. For IHC, after overnight incubation at 4°C with anti-Granzyme B antibody (Bioss, #P10144, 1:50), sections were treated with SP-0023 secondary antibody at room temperature, followed by DAB (#C-0010) chromogenic development.

### Enzyme-linked immunosorbent assay

Serum samples from 24 pLELC patients and 30 healthy controls were analyzed according to manufacturer protocols (**Table 1**, **Supplementary table 1**). TF, mouse IL-10R, and mouse TNF-α levels were quantified using ELISA kits (Elabscience, China).

**Table 1:** Patient characteristics at baseline of pLELC.

Patient characteristics at baseline	DIA discovery cohort *N* (%)	Metabolomics discovery cohort *N* (%)	ELISA cohort *N* (%)
Age			
≤60	4	10	17
>60	1	5	7
Sex			
Male	1	3	10
Female	4	12	14
ECOG performance status			
0–1	4	13	21
>1	1	2	3
Treatment line			
1	4	7	13
>1	1	8	11
TNM			
IIIA-IVA	2	9	15
IVB	3	6	9
Best overall response			
PR	3	5	9
SD	2	9	14
PD	0	1	1
ORR	3 (60%,14.7- 94.7)	5 (33.3%,10.9- 61.7)	9 (37.5%,19.7-58.0)
DCR	5 (100%,47.8- 100.0)	14 (93.9%,67.7,-99.8)	23 (95.8%,79.2-100.0)

DIA, data-independent acquisition; ELISA, Enzyme-linked immunosorbent assay; ECOG, PR, partial response; SD, stable disease; PD, progressive disease. Confirmed complete and partial responses were assessed by the investigator according to the Response Evaluation Criteria in Solid Tumors, version 1.1.

### Animal experiments

All procedures were approved by the Animal Ethics Committee of Panyu Central Hospital, Guangzhou Medical University and complied with ARRIVE 2.0 guidelines. Male BALB/c nude mice (3-6 weeks old) were obtained from Southern Medical University Laboratory Animal Center and housed under specific pathogen-free (SPF) conditions (5-6 mice/cage, 12-h light/dark cycle). Stratified randomization by body weight assigned mice to either:ω-6 diet group (Lieber-DeCarli liquid diet #710027), Control group (standard diet #710028). Both diets were isocaloric (1.0 kcal/mL) with identical macronutrient distribution: 35% fat, 47% carbohydrate, 18% protein (19, 20).

### PDX model establishment and treatment

Fresh tumor tissues from consented pLELC patients (Nanfang Hospital) were subcutaneously implanted into the right dorsum of nude mice using a trocar (2×2×2 mm³ fragments) (21, 22). Stable passage 3 (P3) models were cryopreserved. Treatments began 24h post-implantation: Tisotumab (human anti-TF antibody; AntibodySystem, France): 4 mg/kg, i.p. weekly (vs. IgG control), WY-14643 (PPARα agonist; APExBIO, USA): 100 mg/kg/day in corn oil, i.p. (vs. corn oil control). Mice were euthanized by CO_2_ asphyxiation at endpoint for tumor measurement.

### Quantitative real-time PCR

Total RNA was extracted with TRIzol (Takara, Japan) and reverse-transcribed using PrimeScript RT kits (Takara). SYBR Premix Ex Taq II (Roche, Switzerland) was used for amplification on a LightCycler 480 system. Primer sequences are listed in **Supplementary table 2**. Relative gene expression was calculated by the 2^-ΔΔCT^ method with GAPDH normalization.

### Western blotting

Equal protein amounts were separated by 10% SDS-PAGE, transferred to membranes, and incubated overnight at 4°C with primary antibodies against PPARα (AF7794; Beyotime, China; 1:1000) or NF-κB (AN365; Beyotime; 1:1000). After 1-h incubation with LI-COR fluorescent secondary antibodies (25°C), bands were visualized using an Odyssey infrared imaging system. Experiments were performed in triplicate.

### Statistical analysis

Continuous variables were analyzed by Student’s t-test (normal distribution) or Mann-Whitney U test (non-normal distribution). Categorical data were assessed by Chi-square test. Overall survival (OS) and progression-free survival (PFS) were evaluated via Kaplan-Meier analysis. All analyses used SPSS 19.0 (IBM, USA), with P < 0.05 considered statistically significant.

## Results

### Proteomic identification and validation of TF

**Figure 1:**
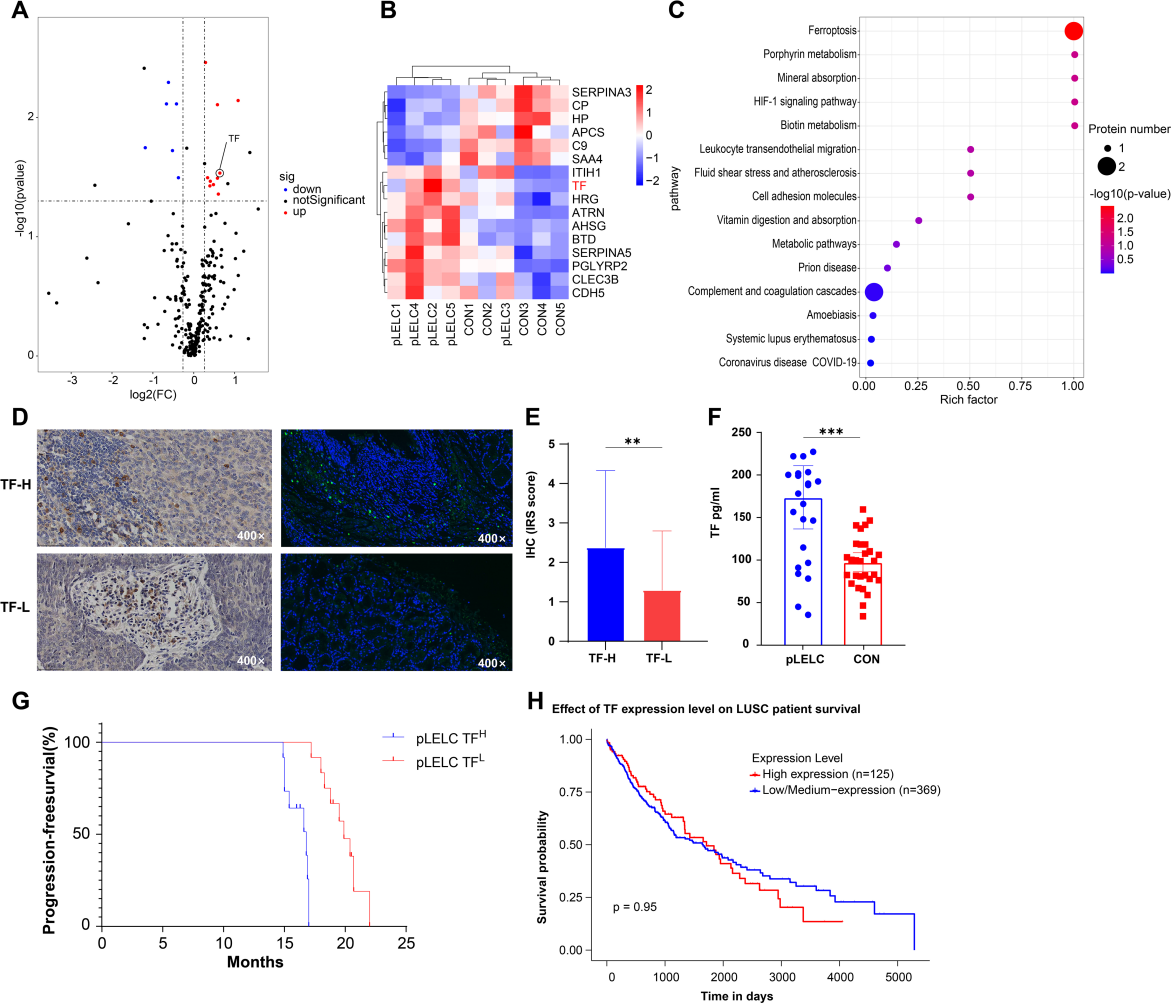
Proteomic identification and validation of TF. **(A)** Volcano plot of differentially expressed proteins (DEPs) in pLELC vs. control (CON) groups. X-axis: log_2_(fold change); Y-axis: -log_10_(P-value). Red dots: significantly upregulated proteins (CV < 0.5, P < 0.05, AVG ≥ 1.2); blue dots: downregulated proteins (CV < 0.5, P < 0.05, AVG ≤ 0.83); black dots: non-significant proteins (P > 0.05). **(B)** Hierarchical clustering heatmap of DEPs. Rows: 16 DEPs (6 downregulated, 10 upregulated); columns: samples (group labels below). Color scale: red (high expression) to blue (low expression), with intensity reflecting magnitude. Clustering used Euclidean distance and Ward’s linkage. **(C)** KEGG pathway enrichment bubble plot of DEPs (top 15 pathways, adjusted P < 0.05). X-axis: Rich factor; Y-axis: pathways (ranked by significance). Bubble color: -log_10_(adjusted P-value) (darker red = higher significance); size: number of DEPs per pathway. **(D)** IF and IHC staining of TF in pLELC tissues, showing strong vs. weak positivity. **(E)** IHC-based immunoreactive score (IRS) of TF in pLELC. High-TF group showed significantly elevated IRS vs. low-TF group (P < 0.05). **(F)** Serum TF levels in pLELC patients vs. healthy controls (ELISA; P < 0.001). **(G)** Progression-free survival (PFS) by TF expression. Kaplan-Meier curve: low-TF group had significantly prolonged PFS (P < 0.0001). **(H)** Overall survival (OS) by TF mRNA expression in TCGA-LUSC cohort (log-rank P = 0.95). ** P<0.01, *** P<0.001.

DIA-based proteomic analysis identified 259 proteins, with 16 exhibiting differential expression between groups (6 downregulated, 10 upregulated; **Supplementary Figure 1**, **Table 2**, **Supplementary tables 3-7**). KEGG pathway enrichment revealed 15 significantly altered pathways, implicating ferroptosis, HIF-1 signaling, metabolic pathways, leukocyte transendothelial migration, and cell adhesion. Ferroptosis demonstrated the highest significance (P = 0.0036), followed by HIF-1 signaling (P = 0.062). TF (P02787) participated in 5 pathways (ferroptosis, HIF-1, mineral absorption). Ceruloplasmin (CP; P00450) associated with ferroptosis and porphyrin metabolism. TF expression was significantly upregulated (1.55-fold, P < 0.05) in pLELC versus controls (**Figures 1A**) and localized to the most enriched pathways. Previous studies indicate TF-targeted therapy reduces M2 tumor-associated macrophage (TAM) infiltration (23), suggesting TF may regulate M2 TAMs in pLELC. IHC confirmed TF positivity (immunoreactive score IRS ≥1) in 87.5% (21/24) of pLELC samples (**Figure 1**). Serum TF levels were significantly elevated in pLELC patients versus healthy controls (**Figure 1**, **Supplementary table 8**). Patients stratified by ELISA-based TF expression revealed prolonged progression-free survival (PFS) in the high-TF group (**Figure 1**). Analysis of TCGA lung squamous cell carcinoma (LUSC) data showed no correlation between TF mRNA levels and overall survival (OS; P = 0.95, **Figure 1**; http://ualcan.path.uab.edu/cgi-bin/TCGA-survival1.pl?genenam=TF&ctype=LUSC).

**Table 2:** Identification of plasma proteome profiles as potential efficacy biomarkers for pLELC.

Protein	pLELC	NOC	Fold change	P value
ATRN	915438.6	434074.425	2.108943875	0.007232952
CP	156394140.8	100992843.2	1.54856657	0.029309541
HP	457536.2688	303437.8031	1.507842016	0.044046333
SERPINA3	2755522.95	1854724.725	1.485677585	0.007827108
APCS	529892.525	356670.7031	1.485663163	0.032368801
C9	8860281.6	6380032.8	1.388751732	0.036641473
AHSG	24628879.2	18698623.6	1.317149311	0.034297511
TF	749988.875	573778.925	1.307104256	0.037533467
HRG	7825187.4	6223416.6	1.257378045	0.032028094
SERPINA5	1371449.5	1130521.25	1.213112535	0.003461194
CLEC3B	25654605.6	33463400	0.766646713	0.032041774
ITIH1	25569924.8	34484727.6	0.74148548	0.007702156
CDH5	6967165.3	10061159	0.692481383	0.019014349
SAA4	6169134.9	9528187.1	0.647461562	0.005088764
BTD	8636641.3	13802516.6	0.625729463	0.007700173
PGLYRP2	201612670.4	461501824	0.436862131	0.017988102

### Metabolomic profiling

**Figure 2:**
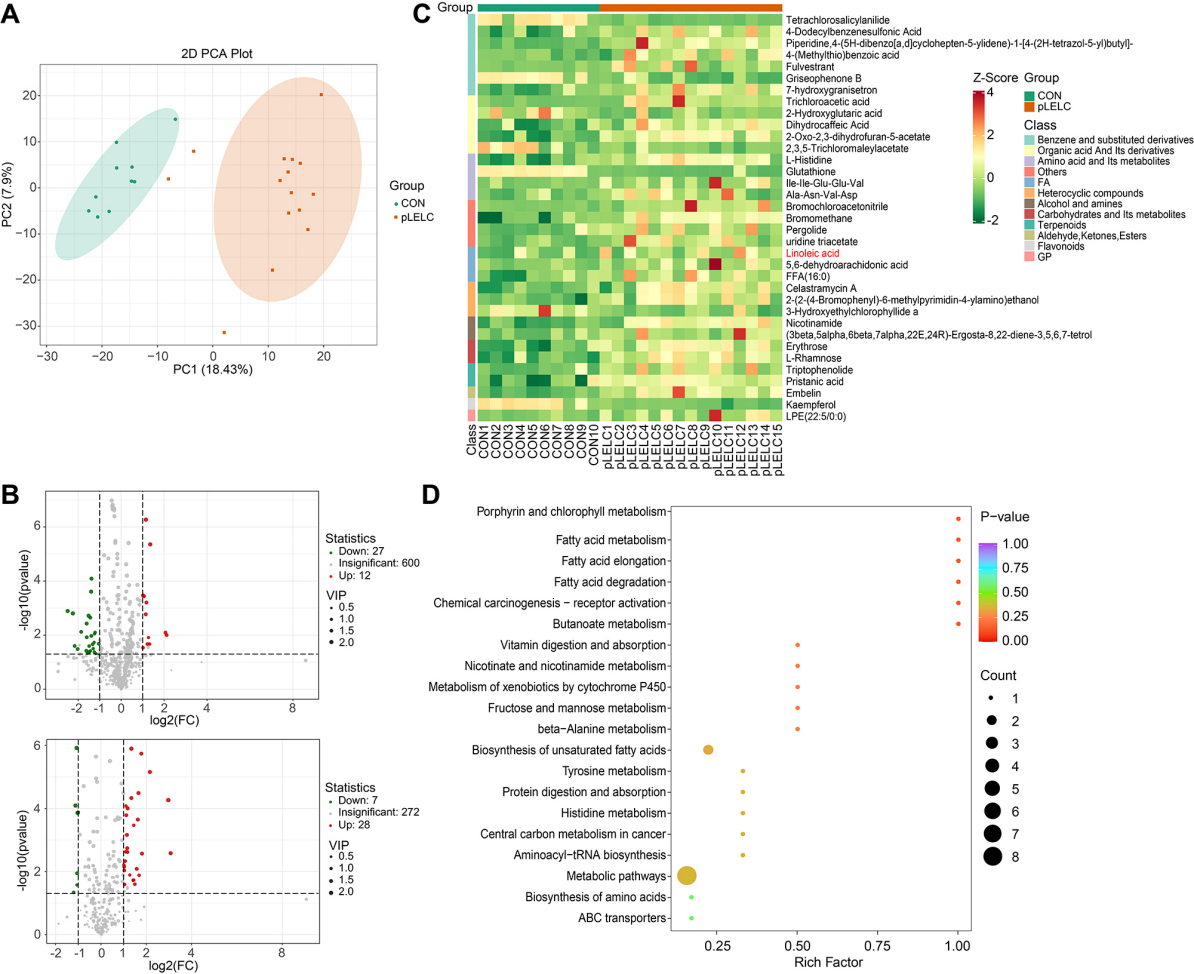
Metabolomic profiling results. **(A)** PCA plot (negative ion mode). PCA of metabolomic data shows separation between pLELC (orange) and control (CON, green) groups along PC1 (18.43% variance) and PC2 (7.9% variance). Ellipses denote 95% confidence intervals. Significant intergroup distinction (PERMANOVA, P = 0.008) indicates global metabolic profile differences. **(B)** Volcano plots of differential metabolites. Top: Positive ion mode; Bottom: Negative ion mode. X-axis: log_2_(fold change); Y-axis: -log_10_(P-value); dot size: VIP score. Red: Significantly upregulated metabolites (VIP ≥ 1, P < 0.05, FC ≥ 2); Green: Downregulated metabolites (VIP ≥ 1, P < 0.05, FC ≤ 0.5); Gray: Non-significant metabolites. **(C)** Hierarchical clustering heatmap of negative ion differential metabolites (n=35). Rows: Metabolites; Columns: Samples. Color scale: UV-scaled relative abundance (red: high; green: low). Key metabolites (e.g., linoleic acid) show significant upregulation in pLELC. **(D)** KEGG enrichment bubble plot of negative ion metabolites (top 20 pathways, adjusted P < 0.05). X-axis: Rich factor; Y-axis: Pathways (significance-ranked). Bubble color: -log_10_(adjusted P) (darker red = higher significance); Size: Number of enriched metabolites.

Untargeted metabolomics detected 3,175 metabolites (946 secondary metabolites), with 74 differentially expressed (34 downregulated, 40 upregulated; **Supplementary tables 9-12**). KEGG analysis enriched 23 pathways, most notably linoleic acid metabolism (ko00591) and unsaturated fatty acid biosynthesis (ko01040), involving key metabolites linoleic acid (LA; C01595) and palmitic acid (C00249) (**Figures 2A**, **Supplementary tables 9-12**). Given established links between ω-6 polyunsaturated fatty acids (PUFAs; particularly LA) and tumor immune microenvironments (24), we focused subsequent experiments on LA-TF interactions.

### TF inhibitor Tisotumab reverses LA-driven pLELC progression

**Figure 3:**
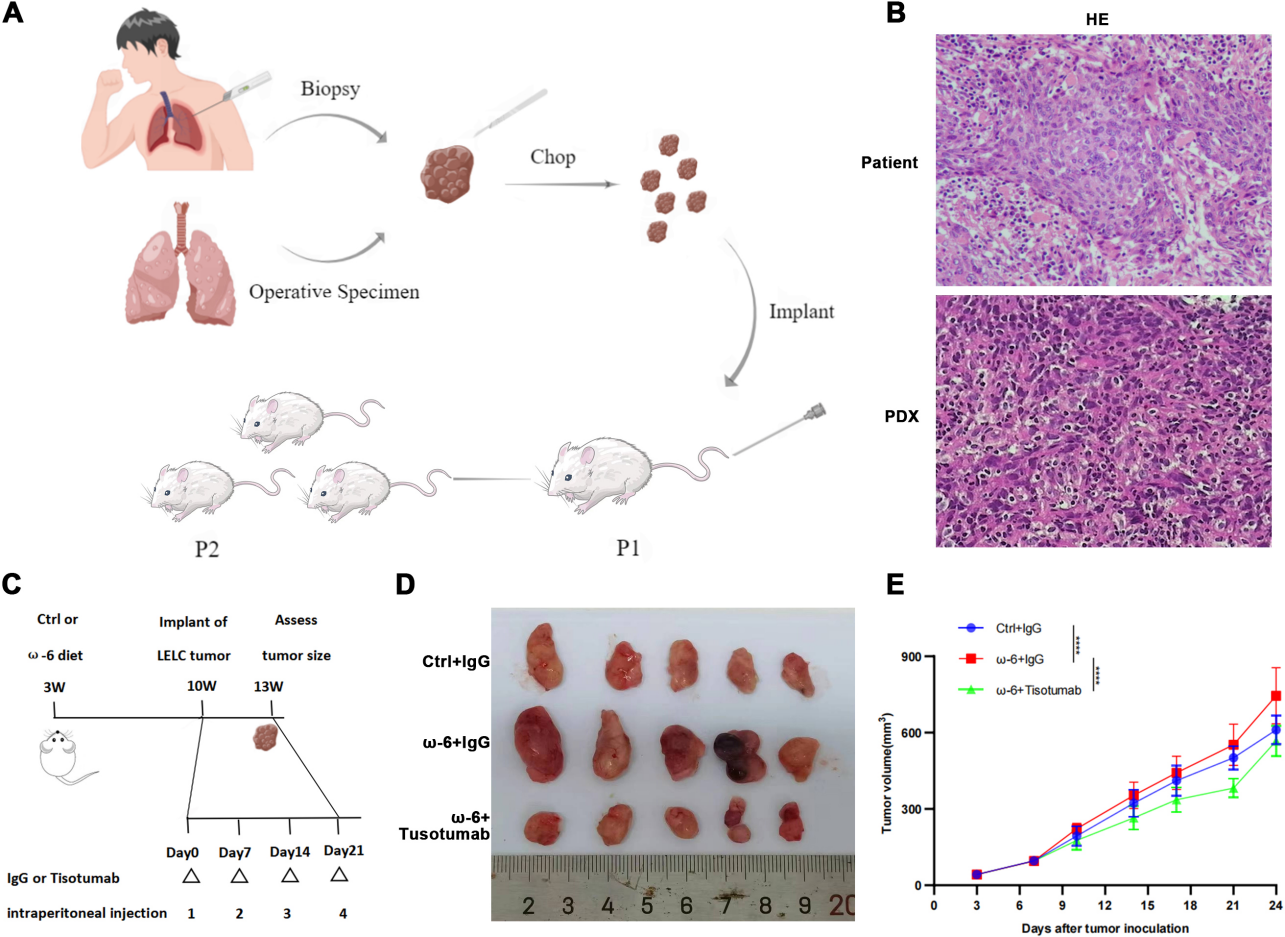
Tisotumab reverses LA-driven pLELC progression. **(A)** PDX model establishment. Tumor tissues from surgery/biopsy were dissected into 2-mm³ fragments and subcutaneously implanted into nude mice via trocar. Stable models were confirmed at passage 3 (P3). **(B)** H&E validation of PDX model. Primary patient tumors and PDX grafts show identical histopathological features. **(C)** Experimental timeline. Mice received ω-6 or control diets from 3 weeks of age. PDX tumors implanted at week 10; Tisotumab (4 mg/kg i.p.) or IgG administered weekly. Tissues harvested at Day 24. **(D)** Macroscopic tumor morphology at endpoint (Day 24). **(E)** Tumor growth curves. ω-6 diet + IgG group exhibited significantly larger volumes vs. control (P < 0.0001), while Tisotumab suppressed ω-6 diet-induced growth (P < 0.0001; two-way ANOVA).

**Figure 4:**
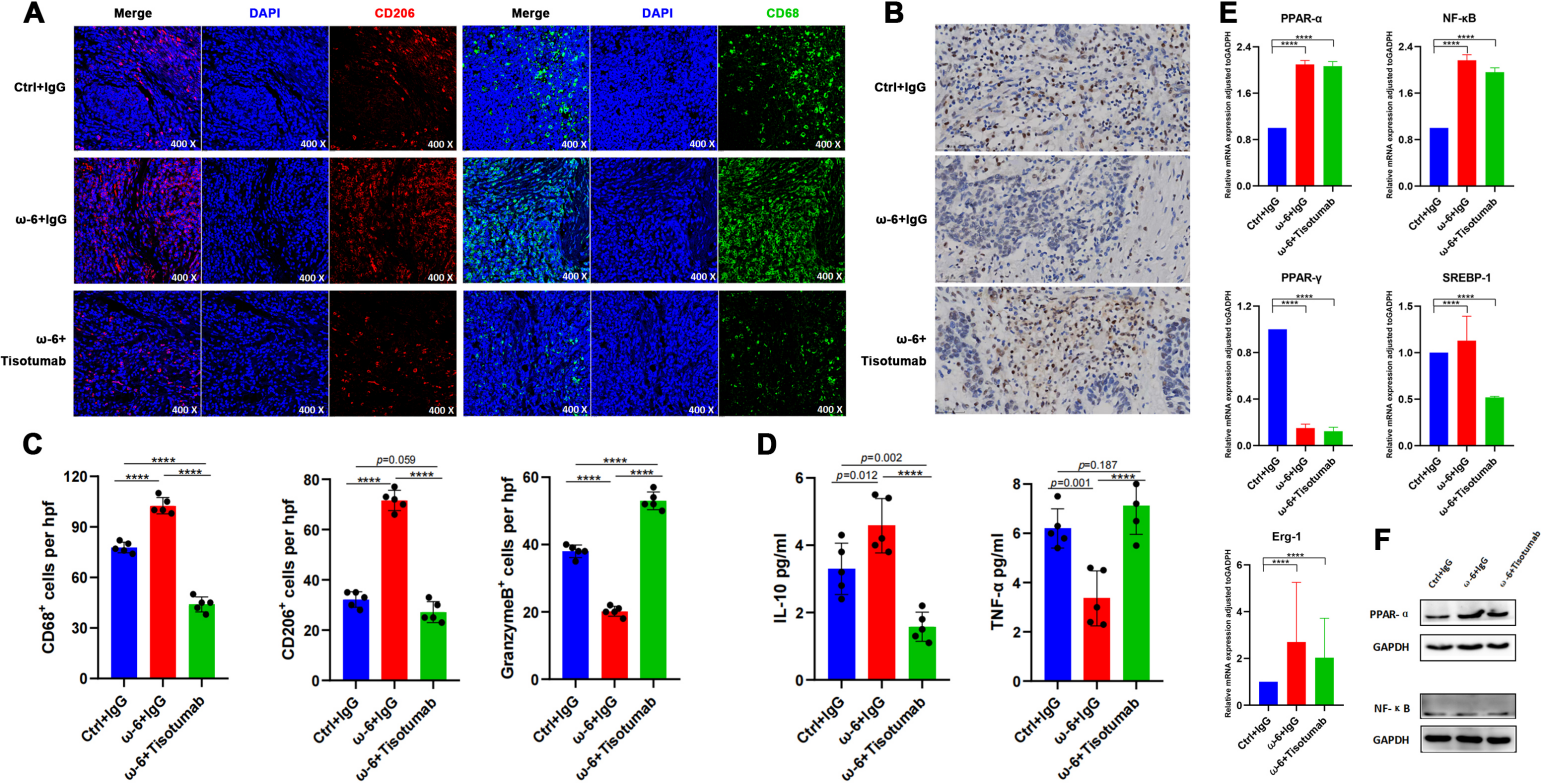
LA promotes M2 TAM infiltration and suppresses NK cells via PPAR-α. **(A)** Representative IF staining of CD68 and CD206 in PDX tumors across groups. **(B)** IHC for Granzyme B in PDX tumors. **(C)** Immune cell quantification. Versus ω-6 diet + IgG group, control and Tisotumab groups showed reduced CD68+, CD206+ cells and increased Granzyme B+ cells (all P < 0.0001). **(D)** Cytokine levels. IL-10 decreased (P = 0.012/P < 0.0001) while TNF-α increased (P = 0.001/P < 0.0001) in control and Tisotumab groups. **(E)** qRT-PCR analysis of transcription factors. ω-6 diet upregulated PPAR-α/NF-κB and downregulated PPAR-γ (all P < 0.0001). No changes in SREBP-1 or Erg-1 (P > 0.05). **(F)** Western blot validation: ω-6 diet significantly increased PPAR-α protein (P < 0.001) without altering NF-κB. **** P<0.0001.

Due to the absence of established pLELC cell lines, PDX models were generated from surgical specimens. H&E staining confirmed histopathological fidelity between PDX tumors and primary tissues (**Figures 3A**). LA (a major ω-6 PUFA) is metabolized by cyclooxygenase (COX)/lipoxygenase (LOX) to arachidonic acid (AA) and derivatives (prostaglandins, leukotrienes) (25, 26). Mice (n= 5) were stratified into:Control (standard diet), ω-6 diet, ω-6 diet + Tisotumab (anti-TF antibody, 4 mg/kg i.p. weekly) group (**Figure 3**). Tumors in the ω-6 diet + IgG group exhibited significantly larger volumes versus control (P < 0.0001). Tisotumab treatment markedly suppressed ω-6 diet-induced tumor growth (P < 0.0001; **Figures 3D**). Immunoanalysis revealed reduced CD68+, CD206+ M2 TAMs and increased Granzyme B+ NK cells in control and Tisotumab groups versus ω-6 diet + IgG (**Figures 4A**). Cytokine profiling showed decreased IL-10 and elevated TNF-α in intervention groups (**Figure 4**).

### LA promotes M2 TAM infiltration and suppresses NK cells via PPAR-α

Experimental data confirm that LA-enriched diets promote M2 TAM infiltration and suppress NK cell activity—effects reversed by TF inhibition. PPARs, sterol regulatory element-binding proteins (SREBPs), and nuclear factor-κB (NF-κB) are established transcriptional regulators of fatty acid metabolism (27–29). Among PPAR isoforms, only PPAR-α and PPAR-γ serve as natural receptors for LA. Quantitative analysis revealed significantly elevated PPAR-α and NF-κB expression in the ω-6 diet + IgG and Tisotumab intervention groups versus controls (P < 0.0001), alongside reduced PPAR-γ. No intergroup differences were observed in SREBP-1 or early growth response factor 1 (Erg-1) expression (**Figure 4**). Western blotting further validated ω-6 diet-induced PPAR-α upregulation without significant NF-κB modulation (**Figure 4**).

### TF inhibitor antagonizes PPAR-α agonist-driven immune remodeling

**Figure 5:**
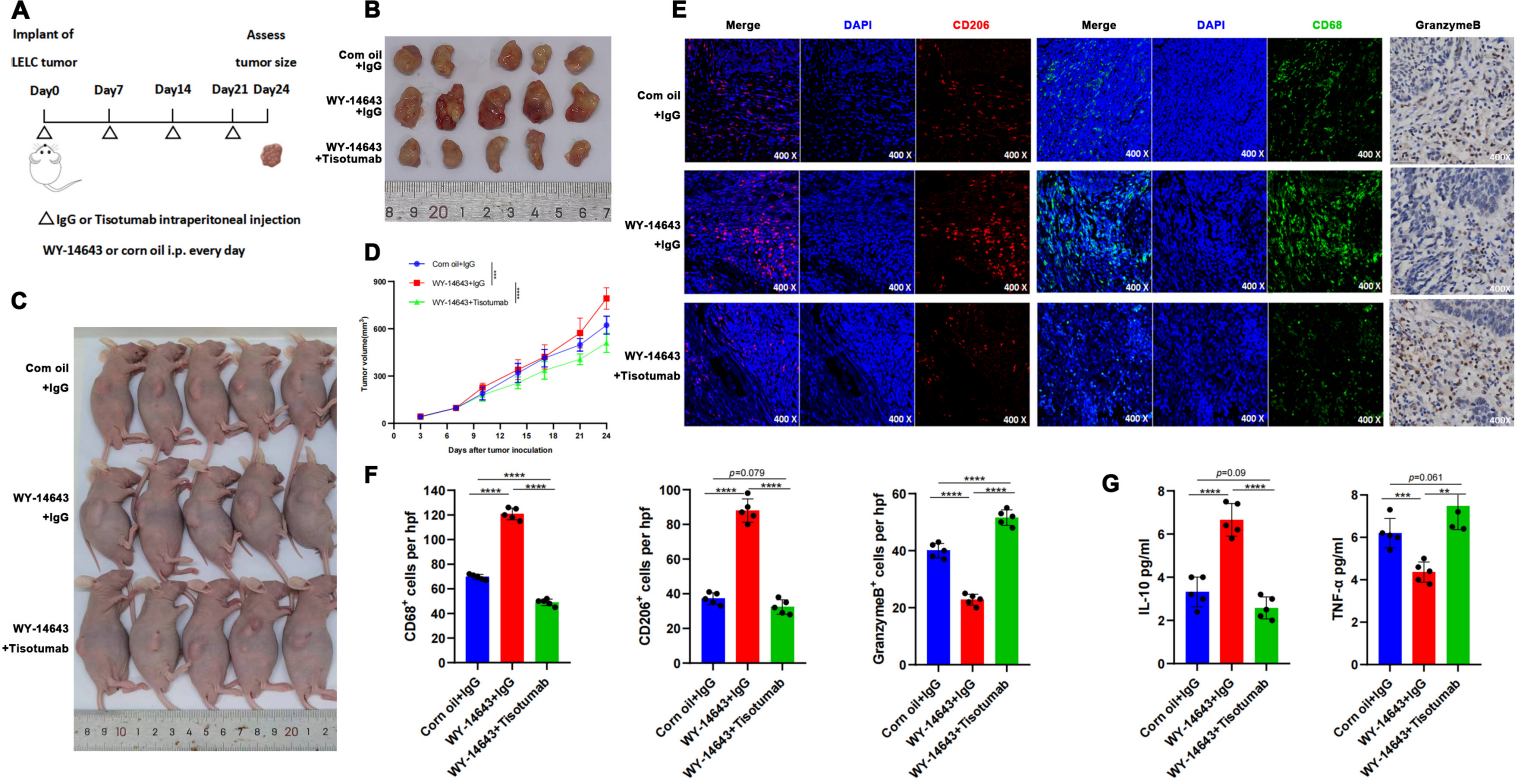
TF Inhibitor reverses PPAR-α agonist-induced immune remodeling. **(A)** Timeline and treatment schedule for umor implant and assessment. PDX inoculation at 6 weeks; daily i.p. WY-14643 (100 mg/kg in corn oil) or vehicle; weekly Tisotumab or IgG; harvest at Day 24. **(B)** Tumor samlpes shown with varying sizes. (Day 24). **(C)** Schematic of mouse endpoint procedures. **(D)** Tumor growth curves: WY-14643 + IgG > control (P < 0.0001); Tisotumab reversed tumor growth (P < 0.0001). **(E)** Microscopic images displaying stained tumor sections at 400x magnification, focusing on CD206, CD68, and GranzymeB markers. **(F)** Bar graphs illustrating the number of CD68+, CD206+, and GranzymeB+ cells per high power field. Control and Tisotumab groups showed reduced CD68+, CD206+ cells and increased Granzyme B+ cells vs. WY-14643 + IgG (all P < 0.0001). **(G)** Bar graphs showing IL-10 and TNF-a levels in various treatments: reduced IL-10 (P < 0.0001) and increased TNF-α (P = 0.004/P < 0.0001) in control and Tisotumab groups. ** P<0.01, *** P<0.001, **** P<0.0001.

Fifteen 6-week-old nude mice (n= 5/group) were allocated to: PPAR-α agonist WY-14643 (100 mg/kg/day i.p.), Corn oil vehicle control, WY-14643 + Tisotumab (**Figure 5**). Tumors in the WY-14643 + IgG group exhibited significantly larger volumes than controls (P<0.0001), while Tisotumab co-treatment suppressed WY-14643-induced tumor growth (P<0.0001; **Figures 5B**). Immunohistochemistry demonstrated reduced CD68+, CD206+ cells and increased Granzyme B+ cells in control and combination groups versus WY-14643 monotherapy (**Figures 5E**). ELISA confirmed decreased IL-10 and elevated TNF-α in the combination group (**Figure 5**).

## Discussion

Tissue factor, a transmembrane receptor and cofactor for FVII/FVIIa, is expressed by pericytes (e.g., adventitial fibroblasts) and surface-lining cells (e.g., epithelial cells). Beyond its central role in hemostasis and thrombosis (30, 31), TF overexpression in tumors correlates with poor prognosis and promotes growth/metastasis (31). In EGFR-mutant NSCLC and glioblastoma, elevated TF predicts adverse outcomes (23). Targeting mTOR and TF remodels the tumor microenvironment by reducing fibrin deposition (ameliorating hypercoagulability), altering collagen distribution (attenuating stromal fibrosis), decreasing CD31+, α-SMA+ vessel density, and diminishing CD206+, F4/80+ immunosuppressive M2 TAM infiltration. Studies in C57BL/6-derived TF-overexpressing tumor cells reveal that TF suppresses NK cell-mediated micrometastasis clearance through fibrinogen- and platelet-dependent mechanisms (32). Additionally, TF promotes metastasis via thrombin-dependent pathways independent of NK cells (32), with recent evidence implicating the TF-thrombin axis in macrophage recruitment for metastatic progression (33). Collectively, TF drives disease progression by remodeling the tumor microenvironment.

Our KEGG analysis highlighted significant ferroptosis pathway enrichment. Key proteins TF and CP may trigger lipid peroxidation by inhibiting GPX4 and enhancing iron toxicity. Although HIF-1 signaling did not reach strict significance (P=0.062), its interplay with ferroptosis suggests hypoxia-mediated regulation of oxidative stress. Future studies should validate direct mechanisms of the TF-CP axis in ferroptosis.

Untargeted metabolomic analysis was performed to elucidate the role of metabolites in pLELC pathogenesis. Results revealed significant enrichment of LA and palmitic acid in unsaturated fatty acid biosynthesis and linoleic acid metabolism pathways. LA may generate pro-inflammatory lipids (e.g., PGE2) via ω-6 PUFA pathways, while palmitic acid supports tumor growth through CD36-mediated lipid uptake. Targeting these metabolic axes—such as combining COX-2 inhibitors with CD36 blockade—may represent novel therapeutic strategies for pLELC.

Accumulating evidence demonstrates distinct roles of polyunsaturated fatty acids (PUFAs) in tumorigenesis: ω-3 family members (α-linolenic acid, EPA, DHA) exert anti-tumor effects, whereas ω-6 PUFAs (LA, AA) exhibit pro-tumor properties (34). Notably, LA demonstrates context-dependent duality—promoting BT-474 and A549 cell proliferation *in vitro* (35). Epidemiologically, high LA intake paradoxically correlates with reduced cancer risk in certain malignancies (36).

Adipose tissue deposition in obesity promotes M2 macrophage polarization, contrasting with M1-dominant profiles in lean individuals (37, 38). Metabolized ω-6 PUFAs generate arachidonic acid (AA), which COX/LOX enzymes convert to pro-inflammatory mediators (prostaglandins, leukotrienes) (39). Crucially: COX-derived PGE-2 induces dendritic cell tolerance and Treg activation (39), AA metabolites (LTB4, LXA4) stimulate expansion of myeloid progenitors (MDSCs, M2 macrophages) (40). Activated M2 macrophages secrete IL-10 to promote Th2 differentiation and PD-L1 upregulation, accelerating T-cell apoptosis and establishing a pro-tumor immunosuppressive feedback loop (40).

TF and LA cooperatively regulate immune equilibrium, particularly M2 macrophage/NK cell/T-cell balance. HGF/c-Met and EGFR pathways upregulate TF expression by activating JNK/Src, PI3K/Akt/mTOR, and KRAS/Raf/MEK/ERK cascades, thereby inducing AP-1, NF-κB, and Egr-1 transcription factors (30). As PPARs and SREBPs constitute master transcriptional regulators of lipid metabolism, this study demonstrates that PPAR-α potentiates LA-induced TF expression. Critically, TF inhibitors effectively reverse PPAR-α agonist-driven tumor progression.

Current mechanistic studies of pLELC primarily focus on genomic and transcriptomic profiles, revealing similarities to nasopharyngeal carcinoma (NPC)—including driver mutations in NF-κB, CDKN2A, and JAK/STAT pathways, analogous regulation of p53 and PD-L1, and shared type II latency features evidenced by LMP1/LMP2 expression (15, 41). Molecular parallels between EBV-positive NPC and pLELC provide rationale for combined therapeutic strategies in advanced disease (42), though pLELC differs significantly from other lung cancers, NK/T-cell lymphoma, or EBV-associated gastric carcinoma (15). First-line treatment for advanced pLELC typically combines immunotherapy or anti-angiogenesis therapy with chemotherapy (43, 44), yet evidence remains limited to small retrospective studies, warranting further exploration of targeted/immunotherapeutic approaches. Our study pioneers multi-omics investigation (proteomics/metabolomics) to identify pLELC vulnerabilities, uncovering a novel LA/PPAR-α/TF axis that drives tumor progression—revealing actionable therapeutic targets.

Beyond our findings, emerging evidence suggests broader regulatory roles. Conjugated linoleic acid (CLA) activates PPAR-γ to alleviate neuroinflammation and promote remyelination (45). Low LA/α-linolenic acid ratios modulate lipid metabolism via PPAR-α/ACOX1 upregulation and SREBP-1c/FAS downregulation (46). CLA induces endogenous PPARα ligands (palmitoylethanolamide/oleoylethanolamide) to exert anti-neuroinflammatory effects (47). Maternal CLA supplementation regulates fetal hepatic lipid metabolism via AMPK signaling (48). Outstanding questions requiring further investigation: Precise mechanisms of LA-mediated TF regulation, TF-PPAR-α feedback dynamics, potential PPAR-α/NF-κB-independent pathways for Tisotumab’s antitumor effects.

Study Limitations:1.No integrated multi-immune-marker prognostic model established. 2.Insufficient fresh tissues for genomic/transcriptomic validation (pLELC rarity). 3.Absence of stable cell lines precluded *in vitro* pathway validation. 4.Modest animal cohort size (n=5) necessitates larger validation. 5.NSG/NSG-SGM3 humanized models unexplored for T/B-cell immunity. 6.Stromal components (e.g., angiogenesis) not analyzed. 7.EBV-LA/TF/PPAR-α axis crosstalk unexamined. Nonetheless, this work provides foundational insights into pLELC immunopathology.

## Conclusion

This study demonstrates that LA promotes pLELC progression via PPAR-α-mediated TF upregulation, identifying TF as a promising therapeutic target. Future directions include: Validating synergistic targeting of the TF-PPARα axis (e.g., Tisotumab + PPARα inhibitor GW6471). Elucidating LA metabolic specificity through gene editing/epigenetic approaches. Clinical translation of TF inhibitor-based combination therapies. Investigating EBV latency protein regulation of TF using patient-derived organoid models.

## Data Availability

The datasets presented in this study can be found in online repositories. The names of the repository/repositories and accession number(s) can be found in the article/**Supplementary Material**.

## Supplementary material

Supplementary Figure 1 Protein electropherograms. **(A)** pLELC group. **(B)** Control (CON) group.

Supplementary Figure 2 LA-TF association. **(A)** Positive correlation between serum LA (μM) and TF (pg/mL) (Spearman r= 0.8536, P = 0.001; n= 15 pLELC patients). **(B)** WB validation: ω-6 diet upregulated TF vs. control; Tisotumab reversed this effect (GAPDH loading control). **(C)** Pre-/post-treatment serum TF: No baseline differences; post-treatment TF in ω-6 + IgG group > control (P < 0.05) and ω-6 + Tisotumab (P < 0.01).

Supplementary Figure 3 NK Cell (NKp46+) infiltration. **(A, B)** ω-6 diet: **(A)** IHC showing reduced NKp46+ cells with ω-6 diet; partial reversal by Tisotumab. **(B)** Quantification: increased NKp46+ cells in control/Tisotumab vs. ω-6 + IgG (all P < 0.001). **(C, D)** WY-14643 treatment: **(C)** IHC showing ↓NKp46+ cells with WY-14643; partial reversal by Tisotumab. **(D)** Quantification: increased NKp46+ cells in control/Tisotumab groups (all P < 0.001).
