# Supplementary figures and images for: Linoleic acid drives pulmonary lymphoepithelioma-like carcinoma progression via PPAR-α/TF axis
Source: Front Oncol. 2025 Aug 15;15:1640201. doi: 10.3389/fonc.2025.1640201 (PMC12394140; doi:10.3389/fonc.2025.1640201)

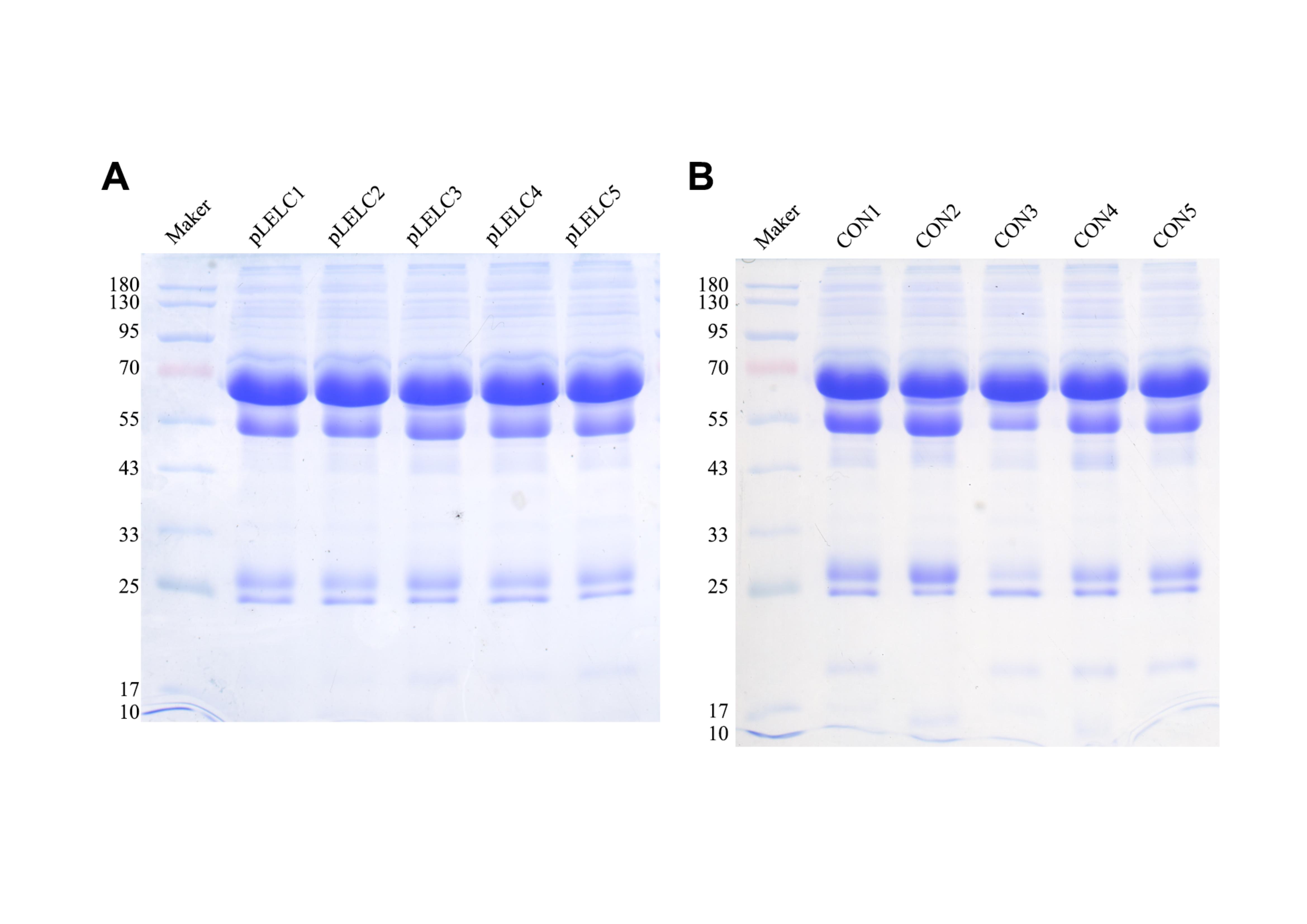

Supplement: Supplementary Figure 1 — Protein electropherograms. (A) pLELC group. (B) Control (CON) group. [file Image1.jpeg]

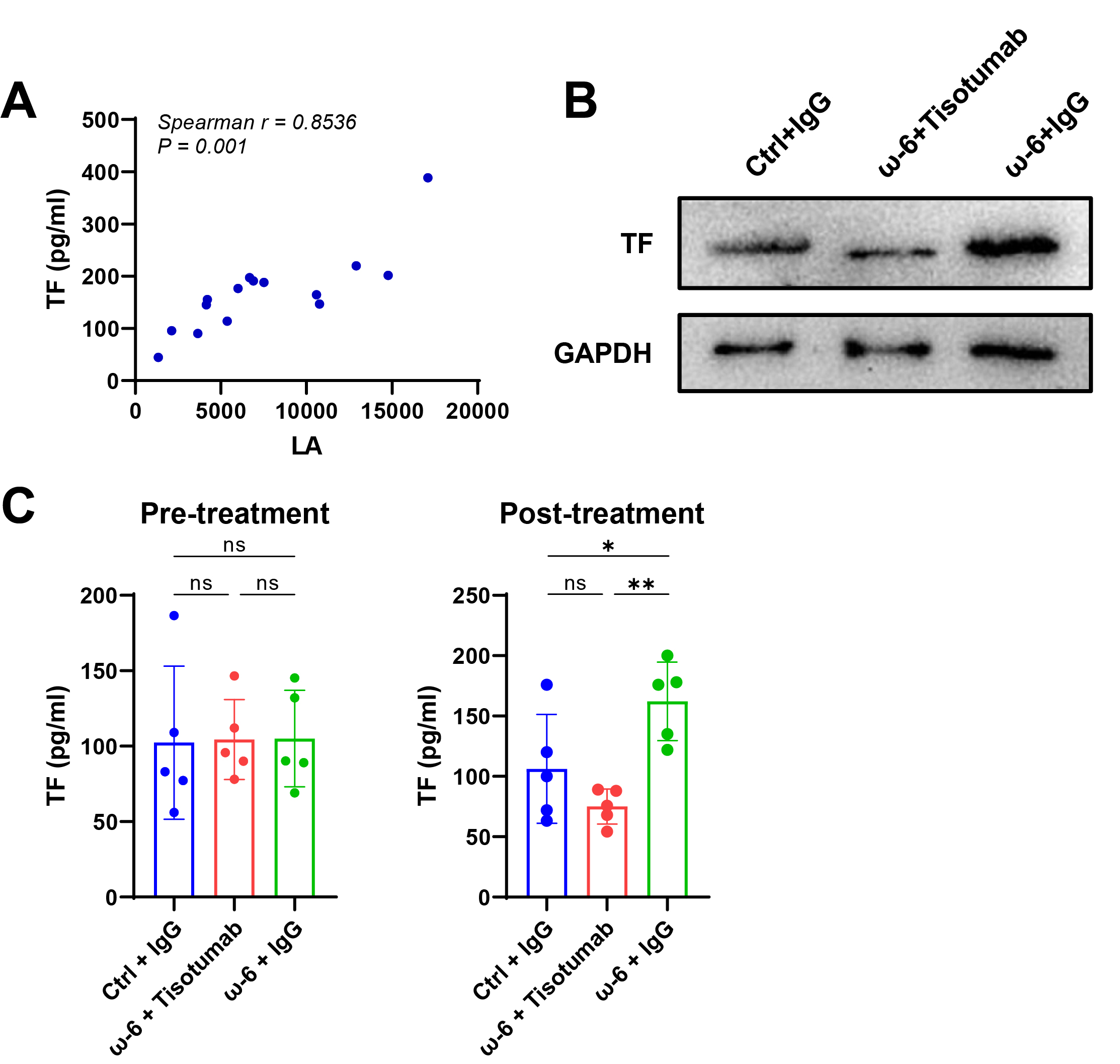

Supplement: Supplementary Figure 2 — LA-TF association. (A) Positive correlation between serum LA (μM) and TF (pg/mL) (Spearman r= 0.8536, P = 0.001; n= 15 pLELC patients). (B) WB validation: ω-6 diet upregulated TF vs. control; Tisotumab reversed this effect (GAPDH loading control). (C) Pre-/post-treatment serum TF: No baseline differences; post-treatment TF in ω-6 + IgG group > control (P < 0.05) and ω-6 + Tisotumab (P < 0.01). [file Image2.jpeg]

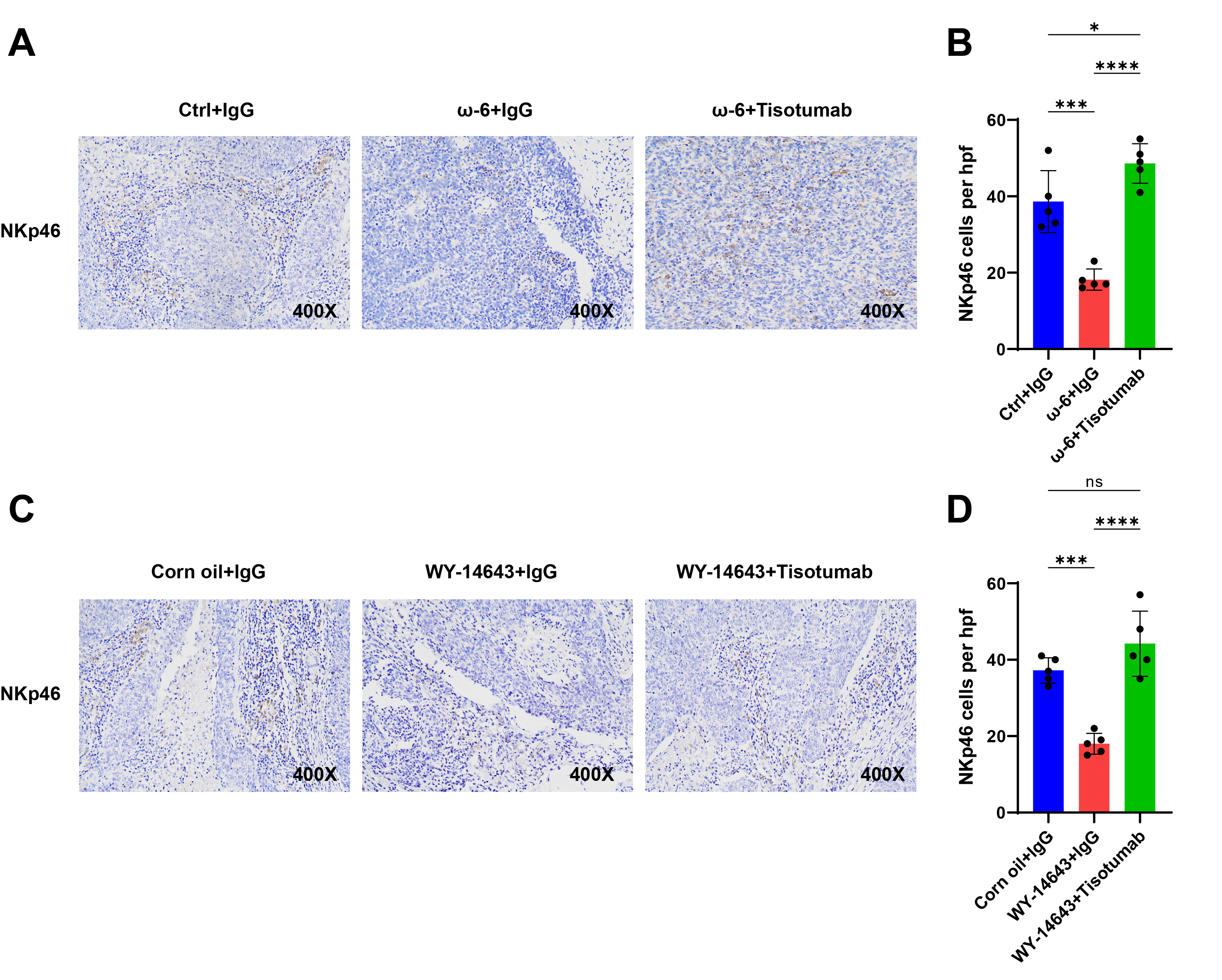

Supplement: Supplementary Figure 3 — NK Cell (NKp46+) infiltration. (A, B) ω-6 diet: (A) IHC showing reduced NKp46+ cells with ω-6 diet; partial reversal by Tisotumab. (B) Quantification: increased NKp46+ cells in control/Tisotumab vs. ω-6 + IgG (all P < 0.001). (C, D) WY-14643 treatment: (C) IHC showing ↓NKp46+ cells with WY-14643; partial reversal by Tisotumab. (D) Quantification: increased NKp46+ cells in control/Tisotumab groups (all P < 0.001). [file Image3.jpeg]
