# Supplementary material for: Linoleic acid drives pulmonary lymphoepithelioma-like carcinoma progression via PPAR-α/TF axis
Source: Front Oncol. 2025 Aug 15;15:1640201. doi: 10.3389/fonc.2025.1640201 (PMC12394140; doi:10.3389/fonc.2025.1640201)
Supplement: Supplementary file 4 [file DataSheet1.zip › Supplementary Table 1.DOCX]

**Supplementary Table 1A.** Detailed treatment history of patients included in proteomics identification.

| Patient ID | Age | Sex | Smoking | ECOG | EBER | Stage at diagnosis | EGFR/ALK | PD-L1 IHC | Drug | Therapy line | Response | irAEs | PFS/months | OS/months |
| --- | --- | --- | --- | --- | --- | --- | --- | --- | --- | --- | --- | --- | --- | --- |
| P1 | 48 | F | No | 0 | Positive | III B | Negative | 30% | Sinyilimab | 1 | SD | Pruritus | 16+ | 16+ |
| P2 | 28 | F | No | 0 | Positive | IV B | Negative | 95% | Sinyilimab | 2 | PR | No | 6+ | 23+ |
| P3 | 20 | M | No | 1 | NA | IV B | Negative | 100% | Sinyilimab | 1 | PR | Rash | 6 | 13 |
| P4 | 62 | F | No | 2 | Positive | IV B | Negative | 80% | Sinyilimab | 1 | SD | No | 2+ | 12+ |
| P5 | 54 | F | No | 1 | Positive | IV A | Negative | NA | Sinyilimab | 1 | PR | No | 3 | 5+ |

NA: not applicable; PR: partial response; SD: stable disease; PD: progressive disease; irAEs: immune-related adverse events.

**Supplementary Table 1B.** Detailed treatment history of patients included in metabolomics identification.

| Patient ID | Age | Sex | Smoking | ECOG | EBER | Stage at diagnosis | EGFR/ALK | PD-L1 IHC | Drug | Therapy line | Response | irAEs | PFS/months | OS/months |
| --- | --- | --- | --- | --- | --- | --- | --- | --- | --- | --- | --- | --- | --- | --- |
| P1 | 48 | F | No | 0 | Positive | III B | Negative | 30% | Sinyilimab | 1 | SD | Pruritus | 16+ | 16+ |
| P2 | 28 | F | No | 0 | Positive | IV B | Negative | 95% | Sinyilimab | 2 | PR | No | 6+ | 23+ |
| P3 | 20 | M | No | 1 | NA | IV B | Negative | 100% | Sinyilimab | 1 | PR | Rash | 6 | 13 |
| P4 | 62 | F | No | 2 | Positive | IV B | Negative | 80% | Sinyilimab | 1 | SD | No | 2+ | 12+ |
| P5 | 54 | F | No | 1 | Positive | IV A | Negative | NA | Sinyilimab | 1 | PR | No | 3 | 5+ |
| P6 | 60 | F | No | 0 | Positive | III B | Negative | 35% | Toripalimab | 2 | SD | Rash | 6+ | 11+ |
| P7 | 62 | M | Yes | 0 | NA | III C | Negative | NA | Sinyilimab | 2 | PD | No | 3 | 30+ |
| P8 | 57 | F | No | 0 | NA | IV A | Negative | NA | Sinyilimab | 2 | SD | Hemoptysis | 4+ | 20+ |
| P9 | 46 | F | No | 1 | Positive | IV B | Negative | 90% | Toripalimab | 1 | SD | No | 8+ | 10+ |
| P10 | 59 | F | No | 0 | Positive | III C | Negative | 90% | Camrelizumab | 1 | PR | No | 5+ | 6+ |
| P11 | 59 | F | No | 1 | Positive | III B | Negative | 30% | Camrelizumab | 3 | SD | No | 3+ | 15+ |
| P12 | 76 | F | No | 2 | Positive | IV B | Negative | 1% | Camrelizumab | 1 | SD | Rash, Pruritus | 3+ | 5+ |
| P13 | 52 | F | No | 0 | Positive | IV A | Negative | 60% | Camrelizumab | 3 | SD | No | 14+ | 38+ |
| P14 | 62 | M | Yes | 0 | Positive | IV B | Negative | NA | Toripalimab | 2 | PR | No | 8 | 37+ |
| P15 | 48 | F | No | 0 | Positive | III B | Negative | NA | Sinyilimab | 3 | SD | Anemia | 8 | 28 |

NA: not applicable; PR: partial response; SD: stable disease; PD: progressive disease; irAEs: immune-related adverse events.

**Supplementary Table 1C.** Detailed treatment history of patients included in ELISA analysis.

| Patient ID | Age | Sex | Smoking | ECOG | EBER | Stage at diagnosis | EGFR/ALK | PD-L1 IHC | Drug | Therapy line | Response | irAEs | PFS/months | OS/months |
| --- | --- | --- | --- | --- | --- | --- | --- | --- | --- | --- | --- | --- | --- | --- |
| P1 | 48 | F | No | 0 | Positive | III B | Negative | 30% | Sinyilimab | 1 | SD | Pruritus | 16+ | 16+ |
| P2 | 28 | F | No | 0 | Positive | IV B | Negative | 95% | Sinyilimab | 2 | PR | No | 6+ | 23+ |
| P3 | 20 | M | No | 1 | NA | IV B | Negative | 100% | Sinyilimab | 1 | PR | Rash | 6 | 13 |
| P4 | 62 | F | No | 2 | Positive | IV B | Negative | 80% | Sinyilimab | 1 | SD | No | 2+ | 12+ |
| P5 | 54 | F | No | 1 | Positive | IV A | Negative | NA | Sinyilimab | 1 | PR | No | 3 | 5+ |
| P6 | 60 | F | No | 0 | Positive | III B | Negative | 35% | Toripalimab | 2 | SD | Rash | 6+ | 11+ |
| P7 | 62 | M | Yes | 0 | NA | III C | Negative | NA | Sinyilimab | 2 | PD | No | 3 | 30+ |
| P8 | 57 | F | No | 0 | NA | IV A | Negative | NA | Sinyilimab | 2 | SD | Hemoptysis | 4+ | 20+ |
| P9 | 46 | F | No | 1 | Positive | IV B | Negative | 90% | Toripalimab | 1 | SD | No | 8+ | 10+ |
| P10 | 59 | F | No | 0 | Positive | III C | Negative | 90% | Camrelizumab | 1 | PR | No | 5+ | 6+ |
| P11 | 59 | F | No | 1 | Positive | III B | Negative | 30% | Camrelizumab | 3 | SD | No | 3+ | 15+ |
| P12 | 76 | F | No | 2 | Positive | IV B | Negative | 1% | Camrelizumab | 1 | SD | Rash, Pruritus | 3+ | 5+ |
| P13 | 52 | F | No | 0 | Positive | IV A | Negative | 60% | Camrelizumab | 3 | SD | No | 14+ | 38+ |
| P14 | 62 | M | Yes | 0 | Positive | IV B | Negative | NA | Toripalimab | 2 | PR | No | 8 | 37+ |
| P15 | 48 | F | No | 0 | Positive | III B | Negative | NA | Sinyilimab | 3 | SD | Anemia | 8 | 28 |
| P16 | 55 | M | No | 0 | Positive | III A | Negative | 30% | Sinyilimab | 1 | PR | No | 6+ | 8+ |
| P17 | 56 | F | No | 1 | Negative | IV B | Negative | 90% | Sinyilimab | 1 | PR | Hypertension | 4+ | 6+ |
| P18 | 44 | M | No | 0 | Positive | III B | Negative | 0 | Sinyilimab | 3 | SD | Tracheal fistula | 7 | 33+ |
| P19 | 52 | M | Yes | 0 | Positive | IV B | Negative | 1000% | Camrelizumab | 1 | PR | No | 2+ | 3+ |
| P20 | 61 | M | Yes | 2 | Positive | IV A | Negative | 95% | Pembrolizumab | 1 | PR | No | 16.3+ | 16+ |
| P21 | 57 | F | No | 0 | Positive | IV A | Negative | 0 | Pembrolizumab | 3 | SD | No | 3 | 20 |
| P22 | 42 | M | Yes | 0 | Positive | III A | Negative | 30% | Sinyilimab | 1 | SD | Rash | 13+ | 13+ |
| P23 | 50 | M | Yes | 1 | Positive | IV B | Negative | NA | Sinyilimab | 3 | SD | No | 3.1+ | 15+ |
| P24 | 67 | M | No | 0 | Positive | III B | Negative | 0 | Tislelizumab | 1 | SD | No | 8.4+ | 10+ |

NA: not applicable; PR: partial response; SD: stable disease; PD: progressive disease; irAEs: immune-related adverse events.
