# Supplementary material for: Linoleic acid drives pulmonary lymphoepithelioma-like carcinoma progression via PPAR-α/TF axis
Source: Front Oncol. 2025 Aug 15;15:1640201. doi: 10.3389/fonc.2025.1640201 (PMC12394140; doi:10.3389/fonc.2025.1640201)
Supplement: Supplementary file 4 [file DataSheet1.zip › Supplementary Table 2.DOCX]

Supplementary Table 2. Primer sequences

| Gene | Primer sequences |
| --- | --- |
| PPAR-α | F: 5′-TGAACAAAGACGGGATG - 3′  R: 5′-TCAAACTTGGGTTCCATGAT - 3′ |
| PPAR-γ | F: 5′-TGAATCCAGAGTCCGCTGACCTC-3′  R: 5′-ATCGCCCTCGCCTTTGCTTTG-3′ |
| NF-κB | F: 5′-ATGTGGAGATCATTGAGCAGC-3′  R: 5′-CCTGGTCCTGTGTAGCCATT-3′ |
| SREBP-1 | F: 5′-GTCTCAGTCCCCTGGTCTCT-3′  R: 5′-TTGATAGGCAGCTTCTCCGC-3′ |
| Erg-1 | F:5′-CGCACAGTGCAGAAAAACG-3′  R: 5′-TGTCTGTTCGGACAACTTCCTT-3′ |
| GAPDH | F: 5′-AGGTCGGTGTGAACGGATTTG-3′  R: 5′-GGGGTCGTTGATGGCAACA-3′ |
